# Characterising the Beyond 50 Cohort: Preliminary Exploration of Physical and Psychosocial Health Factors and Substance Use Among a Cohort of Community Dwelling Adults Aged 50–70 Years

**DOI:** 10.1111/dar.70228

**Published:** 2026-08-09

**Authors:** Rose Laing, Tina Lam, Bosco Rowland, Paul Dietze, Aislinn Lalor, Keith D. Hill, Laura Alfrey, Nadine E. Andrew, Shalini Arunogiri, Stephen Bright, Louisa Picco, Suzanne Nielsen

**Affiliations:** ^1^ Monash Addiction Research Centre, Eastern Health Clinical School, Monash University Melbourne Australia; ^2^ National Centre for Healthy Ageing Melbourne Australia; ^3^ Faculty of Health Deakin University Geelong Australia; ^4^ National Drug Research Institute, Curtin University Melbourne Australia; ^5^ Disease Elimination Program Burnet Institute Melbourne Australia; ^6^ Rehabilitation, Ageing and Independent Living Research Centre, School of Primary and Allied Health Care Monash University Melbourne Australia; ^7^ Department of Occupational Therapy, School of Primary and Allied Health Care Monash University Melbourne Australia; ^8^ Faculty of Education Monash University Melbourne Australia; ^9^ Peninsula Clinical School, School of Translational Medicine Monash University Melbourne Australia; ^10^ School of Medical and Health Sciences Edith Cowan University Perth Australia

**Keywords:** Australia, healthy ageing, mental health, social health, substance use

## Abstract

**Introduction:**

Older adults exhibit unique risks associated with poor mental health and substance use. The Beyond 50 study is a longitudinal cohort investigating the determinants of healthy ageing, including relationships between physical and psychosocial health and substance use. We aim to characterise the Beyond 50 cohort and explore patterns of physical and psychosocial health and substance use.

**Methods:**

Baseline data collection was completed between September 2023 and August 2024. Annual follow‐up data collection commenced in September 2024. Descriptive statistics will be used to characterise the cohort at the time of baseline survey completion.

**Results:**

Of the 1059 participants, the mean age was 61 years (standard deviation 0.2 years) and 46.8% were female. Just over half (52.4%, *n* = 555) received a positive screen for potentially hazardous alcohol consumption (past 12‐month use), 8.7% (*n* = 92) reported recent tobacco use, 3.7% (*n* = 39) reported recent cannabis use and 5.5% (*n* = 58) reported other nonmedical substance use. Twelve percent (*n* = 134) screened positive for the symptoms of moderate to severe anxiety, 16.4% (*n* = 174) screened positive for the symptoms of moderate to severe depression, 15.7% (*n* = 166) were moderately to severely lonely and 22.6% (*n* = 239) reported being dissatisfied with their social relationships.

**Discussion and Conclusion:**

The Beyond 50 cohort displays high rates of alcohol use, with levels of social isolation, loneliness, anxiety and depression comparable to the broader Victoria population. This lends to the cohort's ability to provide further insight into factors that affect healthy ageing and will allow for detailed analysis into the impacts of various physical and psychosocial factors on overall health and wellbeing.

## Key Points Summary

- The Beyond 50 cohort is uniquely placed to uncover insights of healthy ageing and substance use in older Australian adults
- Cohort demographics are broadly consistent with that of the local and national population, supporting the cohort's generalisability to the greater Australian population aged 50–70 years.
- Findings from the Beyond 50 study will directly inform local health policy and harm reduction strategies.

## 1 Introduction

The proportion of the world's population aged 65 years and over has doubled in the last 50 years, rising from 5% in 1970 to 10% in 2023 [1]. By 2030, it is estimated that 1.4 billion people globally (17% of the global population) will be aged 60 years or over, and this is predicted to reach 2.1 billion people by 2050 [2]. In Australia, over 17% of the current population is aged 65 years and over, representing one of the highest proportions of older adults globally [3]. Similar to other high‐income countries such as the United States and United Kingdom, lifestyle‐driven chronic and non‐communicable diseases are now the primary cause of morbidity and mortality [4]. Many of these diseases are preventable and require new approaches to health care provision and disease prevention [5].

In recent years, the concept of healthy ageing has evolved to recognise the influence of socioeconomic factors, psychosocial dynamics and lifestyle choices on health and well‐being [6]. The traditional view of health in later life, once focused primarily on the absence of disease, has expanded to encompass a more holistic perspective. Consequently, since 2015 the World Health Organization (WHO) has defined healthy ageing as an individual's capacity to fulfil essential needs, maintain autonomy, make decisions, engage socially, nurture relationships, stay active, navigate health challenges and disabilities, and live a full life despite illness [7, 8].

While the effects of socioeconomic and demographic factors on healthy aging have already been well documented, other factors such as mental health, social health (which encompasses social isolation and loneliness) and substance use disorders have yet to be fully explored. Older adults are a high‐risk population for mental health conditions, and in Australia one in seven adults aged between 55 and 64 years have an anxiety‐related disorder, while almost one in six have depression or feelings of depression [9], both of which have an adverse impact on quality of life [10]. Poor social health, such as experiences of social isolation and loneliness, have been associated with increased rates of poor mental health, emotional distress, suicide, the development of dementia and premature death [11]. In 2022, 14% of Australian adults aged between 55 and 64 years reported feeling socially isolated [11], and the 2023 Australian ‘State of the Nation’ report found that 34% of adults in the same age bracket were moderately lonely [12]. Finally, substance use disorders in older adults are a growing area of concern. The current population aged 50–70 years have been reported to have riskier drinking and substance use patterns than both previous and younger generations [13]. Reports from the Australian Institute of Health and Welfare reported that around 21% of adults aged 50–59 years smoke tobacco daily and smoke the greatest number of cigarettes per day (16.5 cigarettes) on average than younger or older generations [14]. In addition, one in three adults are estimated to consume alcohol at risky levels (10 or more standard drinks in 1 week), and in the past 10 years recent use of any illicit drug use has doubled among people in their 50's [14]. These rates of substance use leave older adults at a higher risk of associated harms. As such, a deeper understanding of mental health, social health and substance use characteristics is warranted to help support the ageing population.

The Beyond 50 study was established to explore healthy ageing among a cohort of Australians aged 50–70 years, with a focus on outcomes relating to psychosocial health and substance use, considering important factors such as social isolation and loneliness. Findings from this cohort will help inform health prevention strategies to support healthier ageing. This paper aims to describe the characteristics of the cohort and assess its ability to provide insights into factors associated with healthy ageing in adults aged 50–70 years.

### 1.1 Ethics

Ethical approval was granted by the Peninsula Health Human Research Ethics Committee (No. 96756) and by the Monash University Human Research Ethics Committee (No. 35909).

## 2 Methods

### 2.1 Setting and Location

The study is geographically located in the Frankston and Mornington Peninsula local government areas, a southern region of metropolitan Melbourne in Victoria, Australia. This area encompasses some of the highest and lowest areas of socioeconomic disadvantage in Australia [15]. It is also mostly water bound and serviced by Bayside Health Peninsula Care Group (formally Peninsula Health) as the sole public health provider, consisting of four hospitals and over 10 community health and outpatient clinics.

### 2.2 Design

The Beyond 50 study is a longitudinal cohort study of adults aged 50–70 years, with three annual waves of data collection planned. Self‐reported data are collected from participants annually through an online survey, which will be linked with health data, including both health and service utilisation data, from the National Centre of Healthy Ageing Data Platform [16]. The Platform provides comprehensive healthcare data from all public hospitals, community health and outpatient clinics provided by Peninsula Health, with data from 11 different data sets brought together and internally linked within the Peninsula Health data centre [17]. Data linkage will provide 10 years of historical health and health service utilisation data from the date of baseline survey completion, and ongoing health data will be obtained with additional linkages planned after subsequent waves of data collection. Findings are reported following the Strengthening the Reporting of Observational studies in Epidemiology (STROBE) checklist (see Supporting Information A) [18].

### 2.3 Public Involvement

A co‐design consultation phase with input from local service providers and individuals with lived experience (residents from the local study area and aged 50–70 years) was undertaken to identify and provide advice on research priorities and key outcomes of the study, as well as providing guidance on measures of interest and study design. Based on advice from the stakeholders and consumers, a project advisory board was established which provides ongoing advice on the development of ongoing priorities for data analysis. Advisory board members include three ageing specialists, the founder of an assisted living service in the local area, two positive ageing local council representatives and a community member from the target population. The first advisory board meeting was held in February 2024 with an ongoing commitment to meet twice a year, with open correspondence in between when needed. A full description of public involvement can be viewed in Supporting Information B.

### 2.4 Eligibility Criteria

Eligibility criteria included people aged 50–70 years and who live in the Frankston or Mornington Peninsula local government areas and plan to remain residing in the area at the time of recruitment. Participants were required to be cognitively able to complete the survey (self‐reported), and to be either able to read and/or speak English or have someone able to read the survey to them in a language they understand.

### 2.5 Sample Size

A sample size of 1000 participants was calculated to be sufficient to detect differences between high and low levels of social support and loneliness within the cohort. For the Australian population aged 50 years and over, approximately 39% of individuals with depressive symptoms (PHQ‐9 m = 5.2), and 19% without depressive symptoms (PHQ‐9 m = 3.2), have low levels of social support [17, 19]. Power calculations using Stata power two proportion command indicate that with an 80% retention of the original sample (*n* = 800), at a significance level of 0.05, the study has > 95% power to detect this difference in depression between those with high and low levels of social support. Power estimations were calculated by General Linear Mixed Model Power and Sample Size (GLIMMPSE) software.

### 2.6 Recruitment

Cohort recruitment and baseline survey completion occurred between September 2023 and July 2024. Several recruitment methods were utilised to encourage participation from a broad cross‐section of the community and to ensure those typically excluded from research (e.g., due to lack of a home address or internet access) had the opportunity to participate. Recruitment methods included social media advertising (Facebook^TM^), flyer distribution to over 200 local community centres and community gathering areas (including libraries, shopping centres) and mailouts using a gender stratified sampling frame of addresses obtained from the Australian Electoral Commission (randomised, deidentified list of addresses of people aged 50–70 years in the Frankston and Mornington Peninsula local government areas) [20]. A full account on study recruitment has been previously published [21].

### 2.7 Data Collection

Participants completed a baseline survey (first wave of data collection) that gathered information on their general health and wellbeing via their preferred mode of administration. This included online self‐administration or interviewer administration either over the phone or in person, which allowed individuals without access to the internet or smart technology to be included. Seven percent (*n* = 71) of participants opted to complete the survey via phone call or in person. For almost all survey items participants were given the option of ‘prefer not to say’ to enable participants not to answer any questions where they were uncomfortable. All data was collected and managed using REDCap (Research Electronic Data Capture) and hosted and managed by Helix (Monash University) [22].

### 2.8 Variables

Cohort descriptives included in this paper include demographic and socioeconomic characteristics as well as physical health (service utilisation, general health and chronic health conditions), mental health (symptoms of anxiety and depression), social health (social isolation and loneliness) and substance use characteristics that are relevant to the main aims of the study. Where possible, validated scales were used to capture information pertaining to each of these areas. Details on the specific scales and measures and scoring criteria are presented in Table 1. Some variables were collapsed into fewer response options to prevent reporting low response options and protect participant anonymity.

**TABLE 1:** Measures and scoring criteria.

	Domains	Instruments	Scoring/additional details
Demographics	Demographics and socioeconomic status	Individual items from ABS/AIHW [23, 24]	Reported as stand‐alone measures, no scoring required
Physical health	Quality of life	12‐Item Short Form Survey Instrument Version 1 (SF‐12)	Scores are calculated from 12 items covering various aspects of physical and mental health. Responses are weighted and scored using a specific algorithm to generate two summary scores (physical component summary and mental component summary). Scores are standardised with a mean of 50 and a standard deviation of 10 in the general population, with scores above 50 indicating better than average health, while scores below 50 indicate lower than average health.
	Chronic health conditions	Self‐Administered Co‐Morbidity Questionnaire [25]	Reported as stand‐alone measures, no scoring required
	Service utilisation	Healthcare utilisation (6 items)	Reported as stand‐alone measures, no scoring required
	Pain	PEG [26]	A three‐item measure, where each item is scored on a scale of 0–10. The mean score is calculated for the three items by adding the score of each item and dividing the total by three. The higher the score, the greater the pain interference (lowest score 0, highest score 10)
Social health	Social support	Duke Social Support Index [27]	A 10‐item measure used to measure the level of social support. Items 1–4 are scored on the social interaction scale (ranges from 4 to 12), and items 5–11 are scored on the subjective support scale (ranges from 7 to 21). The total DSSI is the sum of both subscales (range 11–33), with higher scores indicating higher social support. Up to two missing values can be imputed as the mean of the remaining values to create DSSI score. Missing values cannot be imputed for subscales.
	Loneliness	UCLA – 4 [28]	A four‐item measure where items are scored to a sum of 4–16, with higher scores indicating higher levels of subjective loneliness.
Mental health	Anxiety	General Anxiety Disorder Assessment (GAD‐7) [29]	Consists of seven items that are scored (range 0–21), with higher scores indicating higher levels of anxiety. One missing value can be imputed as the mean of the remaining values.
	Depression	Patient Health Questionnaire (PHQ‐9) [30]	Consists of nine items that are scored (range 0–27) with higher scores indicating higher levels of depression. Up to two missing values can be imputed as the mean of the remaining values to create.
Substance use	Cannabis usage	CUDIT‐SF [31]	A brief 3‐item measure to screen for cannabis use disorder. Three items are scored (range 0–12), with a score of two or higher considered a positive screen for cannabis use disorder.
	Other drug usage	ASSIST‐lite [32]	Consists of three items for each of: tobacco, opioids, sedatives and stimulants. Three items are scored (range 0–3), with scores of 1–2 indicating moderate risk and scores of three indicating high risk of misuse. A single‐item measure is used for other psychoactive substances (Yes/No indicator).
	Alcohol usage	AUDIT‐C [33]	A brief three‐item measure to screen for hazardous alcohol use. The sum of three items is scored with higher scores indicating a higher likelihood that the patient's drinking is affecting their health and safety (range 0–12). A score of three or more in women and four or more in men is considered a positive screen for hazardous drinking.

*Note:* ABS, Australian Bureau of Statistics; AIHW, Australian Institute of Health and Welfare.

#### 2.8.1 Demographics

Demographic data were collected using single items from the Australian Bureau of Statistics census dictionary [23].

Gender was measured with five response options (i.e., female, male, non‐binary/gender diverse, prefer not to say, prefer to self‐describe) and was recoded into two categories (male and female). All other responses (non‐binary/gender diverse/prefer not to answer and prefer to self‐describe) were coded as missing data to protect participant anonymity due to low representation of other specified genders.

Sexuality was measured with six response options (i.e., straight or heterosexual, lesbian, gay or homosexual, bisexual, queer, other, prefer not to say) and were recoded into two categories (heterosexual and homosexual/bisexual/queer/other).

Relationship status was measured with five response options (i.e., single, married, widowed, separated or divorced, prefer not to say) and was recoded into three categories (single, married/de facto and widowed/separated/divorced).

Work status was measured with 11 response options, where participants could select all that apply (i.e., in paid work, self‐employed, completely retired, partially retired, doing unpaid work/volunteering, studying, managing the household, disabled/sick, unemployed, other, prefer not to say). These were recoded into eight categories (in paid work, self‐employed, completely retired, partially retired, managing the household, disabled/sick, unemployed and other).

Educational attainment was measured with 10 response options (i.e., university degree or higher, certificate/diploma, trade apprenticeship, year 12 or equivalent, year 11 or equivalent, year 10 or equivalent, year nine or equivalent, year eight or below, never attended school, prefer not to say) and were recoded into five categories (university degree or higher, certificate or diploma, trade apprenticeship, year 12 or equivalent and year 11 or below).

Household income was collected and reported as seven response options (i.e., less than A$30,000, A$30,000–$A59,999, A$60,000–A$99,999, A$100,000–A$149,999, A$150,000–A$199,999, A$200,000+, prefer not to say).

Aboriginal and/or Torres Strait Islander descent was collected as five response options (i.e., Aboriginal, Torres Strait Islander, both Aboriginal and Torres Strait Islander, neither and prefer not to say) and were recoded into two categories (Aboriginal and/or Torres Strait Islander and neither).

Current voluntary work status through an organisation and providing other unpaid work/support outside of an organisation was collected and reported as two response options (i.e., yes and no).

Country of birth was collected as 16 response options (i.e., Australia, China, Germany, Greece, Ireland, Italy, Lebanon, Malta, Netherlands, New Zealand, Philippines, Poland, United Kingdom, Vietnam, other (self‐describe), prefer not to say) and were recoded into three categories (Australia, United Kingdom/Ireland and other).

Speaking a language other than English at home was collected and reported as two response options (i.e., yes and no).

#### 2.8.2 Physical Health

A total of 13 chronic health conditions were measured using single items from the Self‐administered Co‐morbidity Questionnaire and included an option to self‐report other chronic health conditions [25]. Participants selected all response options that applied to them.

General health was measured using the SF‐12 (version 1), a 12‐item measure that is scored and weighted using a specific algorithm to create population normalised health scores for both physical and mental health, where the mean health score is 50 with a standard deviation (SD) of 10 [34]. Scores lower than 50 indicate lower than population average physical or mental health, and scores over 50 indicate better than population average physical or mental health. The SF‐12 demonstrated excellent internal consistency (Cronbach's *α* = 0.872).

Medical service utilisation in the last 12 months was measured using a 6‐Item Healthcare Utilisation questionnaire, where participants were asked to self‐report whether they had seen a general practitioner (GP) or medical specialist, or had utilised a hospital emergency department, been admitted to hospital for either physical or mental health, or ambulance service in the last 12 months.

Recent pain and interference with everyday life was measured using the 3‐item Pain, Enjoyment and General Activity scale (PEG) [26]. Items are scored on a scale of 0–10, where 0 indicates no pain and 10 indicates the worst pain imaginable. Scores are summed and the mean of the three items is reported (final score between 0 and 10). The PEG showed excellent internal consistency (*α* = 0.966).

#### 2.8.3 Mental Health

Anxiety was measured using the Generalised Anxiety Disorder instrument (GAD‐7), where seven items are summed to a score of 0–21, and higher scores indicate greater severity of anxiety symptoms. Scores of 0–4 indicate minimal anxiety, 5–9 indicate mild anxiety, 10–14 indicate moderate anxiety and 15–21 indicate severe anxiety [29]. The GAD‐7 showed excellent internal consistency (*α* = 0.906).

Depression was measured using the Patient Health Questionnaire (PHQ‐9) where nine items are summed to a score of 0–27, and higher scores indicate greater severity of depressive symptoms [35]. Scores of 0–4 indicate minimal depression, 5–9 indicate mild depression, 10–14 indicate moderate depression, 15–19 indicate moderate/severe depression and 20–27 indicate severe depression. The PHQ‐9 showed excellent internal consistency (*α* = 0.887).

#### 2.8.4 Social Health

Social isolation was measured using the Duke Social Support Index (DSSI), where 10 items are summed and scored to an overall score of 11–33, and higher scores indicate high levels of social support (low scores indicate social isolation). The DSSI comprises two psychometrically tested subscales. The first four items of the DSSI can be scored to a subscale that represents social interaction with scores between 4 and 12, and the last six items can be scored to a subscale that represents social satisfaction with scores between 7 and 21 [27]. While the social interaction subscale showed moderate internal consistency (*α* = 0.588), this is consistent with prior research [36, 37]. The social satisfaction subscale showed strong internal consistency (*α* = 0.832).

Loneliness was measured using the UCLA Loneliness Scale (UCLA‐LS‐4), where four items are summed to a score of 4–16, and higher scores indicate higher reported loneliness [28]. The recognised cut off score of 11 or higher was used to indicate moderate to severe loneliness [12]. The UCLA‐LS‐4 showed strong internal consistency (*α* = 0.782).

#### 2.8.5 Substance Use

Alcohol use was measured using AUDIT‐C, where three items are scored to a sum of 0–12, and higher scores indicate higher levels of alcohol consumption [33]. A score of 3 or higher in women and 4 or higher in men is considered a positive screen that alcohol consumption may be at levels hazardous to an individual's health [38]. The AUDIT‐C showed acceptable internal consistency (*α* = 0.635) as defined by prior research [33].

Cannabis use was measured using the CUDIT‐SF, where four items are summed to a score of 0–16, and higher scores indicating greater severity of cannabis use disorder. A score of 2 or higher is considered a positive screen for cannabis use disorder [31]. The CUDIT‐SF showed low internal consistency (*α* = 0.456).

Other recent substance use (tobacco, sedatives, stimulants, opioids and other psychoactive substances) was measured using ASSIST‐lite. For each substance, three items are summed to a score of 0–3, with higher scores indicating higher risk of substance use disorder. A score of 0 indicates low risk, 1–2 indicates moderate risk and 3 indicates high risk of substance use disorder [32]. Internal consistency was assessed separately for each ASSIST‐Lite substance subscale, consistent with its design as a set of independent screening domains. The tobacco subscale showed good internal consistency (*α* = 0.682). The sedatives subscale showed low internal consistency (*α* = 0.305). Internal consistency for the stimulants and opioids subscale could not be calculated due to low response rate.

Additional details on the Beyond 50 study design can be found in the protocol paper, which has been prospectively published elsewhere [39].

### 2.9 Analysis

All data analysis was completed using Stata/BE 18.5 [40] and descriptive statistics are reported. Where the data were approximately normally distributed, means and standard deviations are used to describe cohort characteristics. Median scores were reported where data were not normally distributed. The response option ‘prefer not to say’ was treated as missing data for analysis and accounted for all missing data.

Comparisons between key cohort characteristics, including geographical distribution of the cohort, and characteristics of the local and national populations were completed using publicly available statistics derived from the Australian Bureau of Statistics [41, 42] and the Australian Institute of Health and Welfare [24]. Chi‐square goodness‐of‐fit tests were used to assess whether the distribution of sample characteristics differed from those of the broader local and national population.

## 3 Results

**FIGURE 1:**
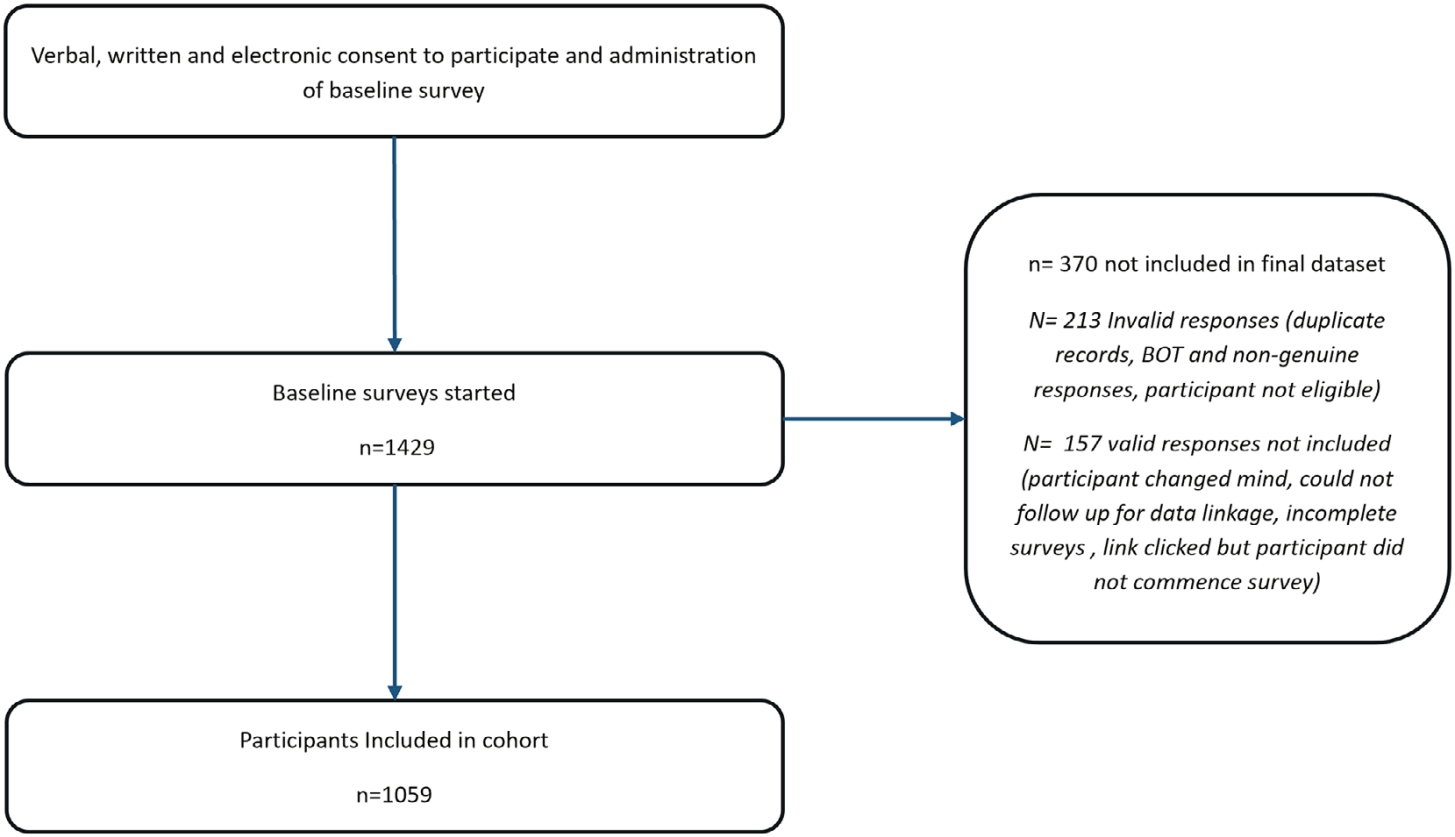
Recruitment flow chart.

In total, the baseline survey was started by 1429 potential participants. Data cleaning resulting in 370 participants being excluded (*n* = 32 incomplete surveys, *n* = 59 duplicate records, *n* = 134 non‐genuine or bot responses, *n* = 20 non‐eligible, *n* = 16 withdrawn participants, *n* = 3 lost to follow up, *n* = 106 survey accessed but no data collected), with a final cohort of 1059 participants (see Figure 1). This resulted in a survey completion rate of 74.1% (valid responses). Just under half of the cohort (*n* = 466, 44%) were recruited through mailouts, with the remaining recruited through other recruitment methods including social media, flyer distribution and newsletters. While exact numbers for mailout recruitment could be accurately calculated due to the use of a unique URL access link, estimates for other methods were inferred through received expression of interest (to social media advertising) with the remainder attributed to flyers/newsletter. Those that were recruited through mailouts were more likely to be married or in a de facto relationship (*χ* ^2^(2) = 45.220, *p* = 0.000), and have higher income (*χ* ^2^(2) = 39.021, *p* = 0.000) than those recruited through other methods; however there was no significant difference in other key demographic characteristics such as educational attainment, retirement status, voluntary work, sexuality, Aboriginal or Torres Strait Islander, or speak a language other than English at home.

### 3.1 Population Density

**FIGURE 2:**
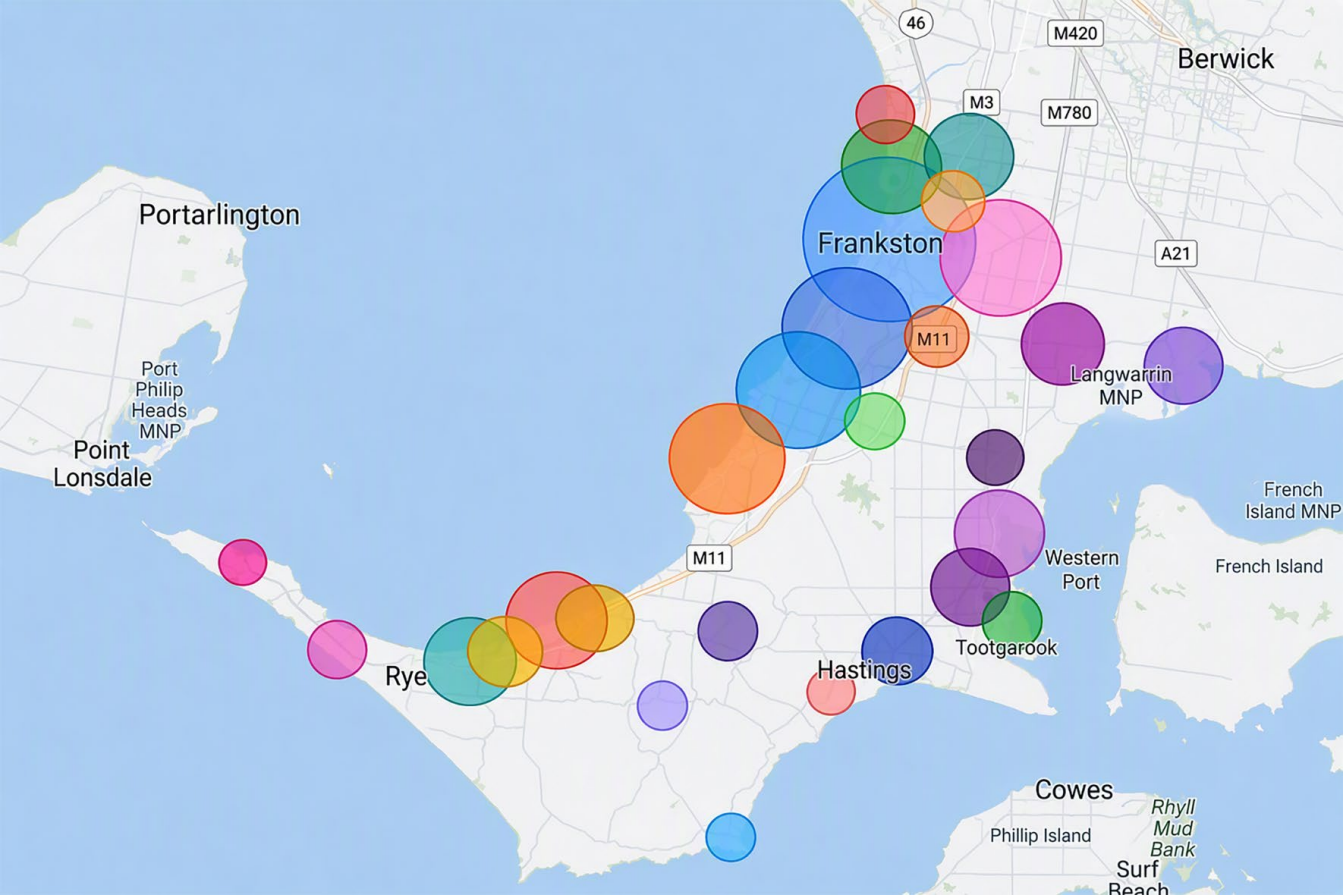
Cohort population distribution by postcode. Geographic distribution of cohort participants across the Frankston and Mornington Peninsula local government areas. The size of each circle is proportional to the number of participants recruited from that location. Different colours are used to visually distinguish overlapping or neighbouring data points and do not represent specific categories or variables.

The final cohort represents individuals from all populated areas within the geographical area, with a greater number of participants located in more densely populated suburbs. Figure 2 shows a map of the cohort population density and spread throughout the Frankston and Mornington Peninsula region. Dots represent density of the cohort population by postcode. When compared to the Australian Bureau of Statistics census data for the region there was less than a 10% difference between the distribution of the cohort compared to the general 50–70‐year‐old population by postcode [41, 42].

### 3.2 Socio‐Demographic Characteristics

Demographic characteristics of the Beyond 50 cohort are presented in Table 2. There were slightly more men than women (52.8% and 46.8%, respectively), with a small number of participants (< 1%) indicating that they identified as non‐binary or gender diverse. The mean age of participants was 61 years (SD = 0.2 years). A small percentage (*n* = 14, 1.3%) identified as being of Aboriginal and/or Torres Strait Islander descent and 7.8% (*n* = 83) reported speaking a language other than English at home.

**TABLE 2:** Baseline sociodemographic characteristics of participants.

	Male, *N* = 559 (%)	Female, *N* = 496 (%)	Total, *N* = 1059 (%)
Age, years			
50–54	109 (19.5)	106 (21.4)	215 (20.3)
55–59	95 (17.0)	98 (19.8)	194 (18.3)
60–64	137 (24.5)	141 (28.4)	281 (26.5)
65–70	218 (39.0)	151 (30.4)	369 (34.8)
Relationship status			
Single	33 (5.9)	82 (16.5)	115 (10.9)
Married/de facto	466 (83.4)	292 (58.9)	761 (71.9)
Widowed/separated/divorced	55 (9.8)	114 (23.0)	169 (16.0)
Sexual identity			
Heterosexual	536 (95.9)	468 (94.4)	1005 (94.9)
Homosexual/bisexual/queer/other	18 (3.2)	22 (4.4)	42 (4.0)
Highest level of educational attainment			
University degree	254 (45.4)	219 (44.2)	476 (45.0)
Certificate/diploma	137 (24.5)	167 (33.7)	305 (28.8)
Trade/apprenticeship	63 (11.3)	6 (1.2)	69 (6.5)
Year 12	51 (9.1)	51 (10.3)	102 (9.6)
Year 11 or below	53 (9.5)	53 (10.7)	106 (10.0)
Current/most recent occupation			
Manager	157 (28.1)	50 (10.1)	207 (19.6)
Professional	168 (30.1)	163 (32.9)	332 (31.4)
Technician/trades worker^a^	—	—	74 (7.0)
Community/personal services worker	18 (3.2)	65 (13.1)	83 (7.8)
Clerical/administrative worker	24 (4.3)	96 (19.2)	119 (11.2)
Sales worker	14 (2.5)	21 (4.2)	36 (3.4)
Machinery operator and driver^a^	—	—	16 (1.5)
Labourer^a^	—	—	18 (1.7)
Other/not applicable	75 (13.4)	92 (18.6)	169 (16.0)
Current work status^b^			
In paid work	252 (45.1)	216 (43.6)	471 (44.5)
Self employed	81 (14.5)	58 (11.7)	139 (13.1)
Completely retired	152 (27.2)	116 (23.4)	268 (25.3)
Partially retired	72 (12.9)	54 (10.9)	127 (12.0)
Managing the household	10 (1.8)	36 (7.3)	47 (4.4)
Disabled/sick	12 (2.3)	21 (4.2)	33 (3.1)
Unemployed	17 (3.0)	8 (1.6)	25 (2.4)
Other	6 (1.1)	15 (3.0)	21 (2.0)
Current voluntary work			
Through an organisation	175 (31.3)	160 (32.3)	336 (31.7)
Unpaid support work (outside of an organisation)	116 (20.8)	138 (27.8)	255 (24.1)
Yearly household income			
< $30,000	48 (8.6)	73 (14.7)	121 (11.4)
$30–59,000	73 (13.1)	110 (22.2)	184 (17.4)
$60–99,000	135 (24.2)	110 (22.2)	256 (23.2)
$100–149,000	100 (17.9)	76 (15.3)	177 (16.7)
$150–199,000	73 (13.1)	39 (7.9)	113 (10.7)
> $200,000	89 (15.9)	26 (5.2)	115 (10.9)
Speaks a language other than English at home			
Yes	45 (8.1)	37 (7.5)	83 (7.8)
No	514 (92.0)	457 (92.1)	974 (92.0)
Indigenous status			
Aboriginal and/or Torres Strait Islander	7 (1.3)	7 (1.4)	14 (1.3)
Neither	544 (97.3)	483 (97.4)	1030 (97.3)
Current housing			
House	491 (87.8)	400 (80.7)	895 (84.5)
Flat/unit/apartment	57 (10.2)	79 (15.9)	136 (12.8)
Retirement village/other	10 (1.8)	16 (3.2)	26 (2.5)

*Note:* Percentage of total cohort with missing data (gender 0.1%, age 0%, relationship status 1.3%, sexual identity 1.1%, highest level of education 0.1%, occupation 0.5%, current work status 0.7%, volunteer work through an organisation 0.7%, provide unpaid work/support to people 0.9%, household income 9.7%, speaks language other than English at home 0.2%, Aboriginal and/or Torres Strait Islander descent 1.4%, current housing 0.2%).

^a^ Values omitted if value represented less than 1% of gendered population.

^b^ Participants were able to select more than one response option where applicable.

### 3.3 Service Utilisation, Physical Health and Functional Capacity

Table 3 reports service utilisation and physical health characteristics of the cohort. The most reported chronic health conditions were high blood pressure (29.8%, *n* = 315), back pain (29.5%, *n* = 312) and depression (15.9%, *n* = 168). One in five (20.6%, *n* = 218) reported no chronic health conditions. Almost a third (29.1%, *n* = 233) of participants reported that their physical health currently limits them when carrying out moderate everyday activities. Approximately three‐quarters (72.5%, *n* = 766) of participants reported experiencing pain in the last week, with the median pain score being 1.33 (min score = 0, max score = 10).

**TABLE 3:** Participant physical and mental health characteristics.

	Male, *N* = 559 (%)	Female, *N* = 496 (%)	Total, *N* = 1059 (%)
Service utilisation in last 12 months			
General practitioner	526 (94.1)	473 (95.4)	1003 (94.7)
Medical specialist	343 (61.4)	309 (62.3)	653 (61.7)
Hospital emergency department	84 (15.0)	97 (19.6)	181 (17.1)
Admitted to hospital for physical health	126 (22.5)	102 (20.6)	228 (21.5)
Admitted to hospital for mental health^a^	—	—	11 (1.0)
Received care from an ambulance	38 (6.8)	41 (8.3)	79 (7.5)
Comorbidities^b^			
Heart disease	69 (12.3)	27 (5.4)	96 (9.1)
High blood pressure	193 (34.5)	122 (24.6)	315 (29.8)
Lung disease	16 (2.9)	22 (4.4)	38 (3.6)
Diabetes	59 (10.6)	32 (6.5)	92 (8.7)
Ulcer or stomach disease	13 (2.3)	22 (4.4)	35 (3.3)
Kidney disease	10 (1.8)	11 (2.2)	21 (2.0)
Liver disease	6 (1.1)	9 (1.8)	15 (1.4)
Anaemia/other blood disease	8 (1.4)	14 (2.8)	22 (2.1)
Cancer	29 (5.2)	20 (4.0)	49 (4.6)
Depression	70 (12.5)	98 (19.8)	168 (15.9)
Osteoarthritis/degenerative arthritis	81 (14.5)	146 (29.4)	228 (21.5)
Back pain	178 (31.8)	133 (26.8)	312 (29.5)
Rheumatoid arthritis	15 (2.7)	21 (4.2)	36 (3.4)
Other medical problem	152 (27.2)	207 (41.7)	360 (34.0)
Pain (in last week)			
Experienced pain	388 (69.4)	375 (75.6)	766 (72.5)
Does participant's health limit moderate everyday activities			
Limited a lot	36 (6.4)	38 (7.7)	75 (7.1)
Limited a little	100 (17.9)	132 (26.6)	233 (22.0)
Not limited	423 (75.7)	325 (65.5)	750 (70.8)
Symptoms of anxiety (last 2 weeks)			
None/minimal	374 (66.9)	272 (54.8)	648 (61.2)
Mild	124 (22.2)	146 (29.4)	272 (25.7)
Moderate	37 (6.6)	44 (8.9)	81 (7.7)
Severe	22 (3.9)	31 (6.3)	53 (5.0)
Symptoms of depression (last 2 weeks)			
None/minimal	377 (67.4)	263 (53.0)	641 (60.5)
Mild	111 (19.9)	128 (25.8)	241 (22.8)
Moderate	36 (6.4)	59 (11.9)	96 (9.1)
Moderate severe	21 (3.8)	29 (5.9)	50 (4.7)
Severe	13 (2.3)	15 (3.0)	28 (2.6)

*Note:* Percentage of total cohort with missing data (general practitioner utilisation 0.5%, medical specialist 1.1%, emergency department 1.3%, admitted for physical health 0.9%, admitted for mental health 0.9%, received care from an ambulance 0.7%, chronic health conditions 0.2%, pain 0.2%, limited in activities 0.1%, symptoms of anxiety 0.5%, symptoms of depression 0.3%).

^a^ Values omitted if value represented less than 1% of gendered population.

^b^ Participants able to select more than one response option where applicable.

General health as measured by SF‐12 showed a mean physical health score of 49.3 (SD = 9.66) where the lowest recorded score was 17.8 and the highest score was 65.4, and a mean mental health score of 49.7 (SD = 10.58) where the lowest recorded score was 11.5 and the highest score was 68.7.

### 3.4 Mental Health

Around one in eight (12.7%, *n* = 134) screened positive for symptoms of moderate to severe anxiety as defined by a GAD‐7 score of 10 or higher. Around one in six (16.4%, *n* = 174) screened positive for symptoms of moderate to severe depression, as defined by a PHQ‐9 score of 10 or higher (see Table 3).

### 3.5 Social Health

The median score for the DSSI social interaction and social satisfaction subscales was 8 (min 4, max 12), and 20 (min 7, max 21), respectively. The overall mean DSSI score was 26.5 (SD = 3.8, min = 11 max = 33). Almost a quarter (22.6%, *n* = 239) of people indicated that they were dissatisfied with their current social relationships.

The mean UCLA‐LS‐4 score for the cohort was 8.3 (SD = 2.4, min = 4, max = 16), with 15.7% (*n* = 166) of the cohort identifying as being moderately to severely lonely.

### 3.6 Substance Use Characteristics

Substance use among the cohort is reported in Table 4. Current alcohol use (past 12 months) was reported by 85.2% (*n* = 902) of participants. A positive screen for hazardous alcohol consumption was reported for over half (52.4%, *n* = 555) of participants. Cannabis use in the past 3 months was reported by 3.7% (*n* = 39) of participants, of which almost a third (*n* = 11) screened positive for cannabis use disorder (as per CUDIT‐SF guidelines), representing around 1.0% of the total cohort [31]. Tobacco use was reported by 8.7% (*n* = 92) of the cohort. Less common substance use reported by cohort members included stimulant use (0.9%, *n* = 10), sedative use (3.7%, *n* = 39) and other psychoactive substances (0.9%, *n* = 9), while no participants reported recent nonmedical opioid use.

**TABLE 4:** Substance use characteristics.

	Male, *N* = 559 (%)	Female, *N* = 496 (%)	Total, *N* = 1059 (%)
Alcohol consumption (AUDIT‐C; last 12 months)			
Current use	495 (88.6)	403 (81.3)	902 (85.2)
Negative screen	182 (32.6)	155 (31.3)	337 (31.8)
Positive screen	312 (55.8)	239 (48.2)	555 (52.4)
Current cannabis use (CUDIT; last 3 months)			
Current use	28 (5.0)	11 (2.2)	39 (3.7)
Current tobacco use (ASSIST‐lite; last 3 months)			
Current use	49 (8.8)	43 (8.7)	92 (8.7)
Other current substance use (ASSIST‐lite; sedative, stimulant, opioid and psychoactive; last 3 months)			
Current use	29 (5.2)	29 (5.9)	58 (5.5)

*Note:* Percentage of total cohort with missing data (current alcohol use 0.3%, AUDITC screen 1.6%, cannabis use 0.3%, tobacco use 0%, other substance use 0%).

### 3.7 Comparison With Local and National Australian Population

Comparisons between cohort demographic characteristics and those of the local and national Australian populations of the same age range can be seen in Table 5. Briefly, the cohort has a comparable gender distribution with the local population (52.8% male in cohort vs. 48.8% male locally, *χ* ^2^(1) = 3.670, *p* = 0.055). Representation of people of Aboriginal and Torres Strait Islander descent was not significantly different from the local population (1.3% in cohort vs. 1.1% locally; *χ* ^2^(1) = 0.156, *p* = 0.693) or the national population (2.1%, *χ* ^2^(1) = 1.733, *p* = 0.188). The cohort exhibited significantly higher rates of being married than both the local and national population (71.9% in cohort vs. 48.3% locally vs. 61.0% nationally; *χ* ^2^(1) = 123.012, *p* < 0.000, *χ* ^2^(1) = 28.000, *p* < 0.000), having a bachelor's degree or higher (45.0% in cohort vs. 22.0% locally vs. 35.5% nationally; *χ* ^2^(1) = 125.193, *p* < 0.000, *χ* ^2^(1) = 17.688, *p* < 0.000), and of volunteering (31.7% in cohort vs. 10.8% locally vs. 15.0% nationally; *χ* ^2^(1) = 141.070, *p* < 0.000, *χ* ^2^(1) = 84.219, *p* < 0.000).

**TABLE 5:** Demographic comparisons of the Beyond 50 cohort compared to local and national characteristics in the same age bracket.

		% of population			
		Beyond 50 cohort	Mornington Peninsula	Frankston	Australia
Gender	Male	52.8	47.7	49.9	48.7
	Female	46.8	52.3	49.9	51.3
Indigenous status	Aboriginal and/or Torres Strait Islander descent	1.3	0.7	1.5	2.1
Country of birth	Australia	76.4	68.4	61.0	62.0
	United Kingdom	13.2	12.3	10.1	8.2
	New Zealand	1.5	2.6	4.1	3.0
Language	Other than English at home	8.1	8.3	11.6	22.5
Relationship status	Married	71.9	58.6	38.0	61.0
	Widowed/separated/divorced	16.0	28.6	40.5	25.5
Work status	Working	44.1	64.4	49.6	61.2
Education	Bachelor's degree or higher	45.0	19.2	24.8	35.5
Volunteer	Voluntary work through an organisation	31.7	13.1	8.4	15.0

*Note:* values for Mornington Peninsula, Frankston and Australia are derived from Australian Bureau of Statistics community profile data for people aged 55–64 years, which uses data collected from the 2021 Census of Population and Housing Australia.

Self‐reported service utilisation was higher than national rates for both GP and specialist use (GP visits 94.7% in cohort vs. 89.3% nationally, *χ* ^2^(1) = 23.350, *p* < 0.000; medical specialist visits 61.7% in cohort vs. 46.3% nationally, *χ* ^2^(1) = 56.126, *p* < 0.000), while self‐reported emergency visits were comparable to national rates (17.9% in cohort vs. 16.9% nationally, *χ* ^2^(1) = 0.039, *p* = 0.844).

Recent alcohol use was shown to reflect nationally reported use for the same age bracket (85.2% in cohort and 84.4% nationally, *χ* ^2^(1) = 0.447, *p* = 0.504). Conversely, use of other substances was low within the cohort compared to national rates in the same age bracket for tobacco (8.7% in cohort vs. 11.7% nationally, *χ* ^2^(1) = 5.279, *p* = 0.022), cannabis (3.7% in cohort vs. 6.2% nationally, *χ* ^2^(1) = 6.759, *p* = 0.009) and illicit substances including stimulants, sedatives and psychoactive (e.g., LSD, psilocybin) (5.2% in cohort vs. 7.8% nationally, *χ* ^2^(1) = 6.077, *p* = 0.014) [43].

## 4 Discussion

We provide an overview of the Beyond 50 cohort, consisting of people aged 50–70 years living in the Frankston and Mornington Peninsula region. While the cohort characteristics are consistent with the broader population of the same age in geographical spread and some demographic characteristics (i.e., gender, identifying as Aboriginal and/or Torres strait Islander, alcohol use) there was variability in some other characteristics (i.e., marital status, other substance use).

Initial exploration of the cohort has revealed a high prevalence of potentially hazardous alcohol use, with over half of the population receiving a positive AUDIT‐C screen. While a positive screen cannot be considered a diagnosis of alcohol use disorder, it does flag that individual assessment may be required to determine if patterns of alcohol use are likely to result in adverse health outcomes [33]. Ageing is associated with metabolic and cognitive changes that affect sleep patterns, increase risk of falls and other injury, and drive cognitive decline, all of which are exacerbated by alcohol use [44]. Given the demographic shift towards an older population in Australia, it is essential to understand not only how alcohol use impacts health outcomes in this population, but also identify correlates, and risk factors for and perceptions of high alcohol consumption. The Beyond 50 cohort is uniquely positioned to track changes in alcohol consumption in this population over time. Longitudinal data will allow us to develop insights into the direct impacts of alcohol use on health outcomes, and understand how other aspects of wellbeing, such as social health, may impact individuals' consumption. These insights will be critical to informing policy and targeted interventions to reduce associated health burden in the Australian ageing population.

Within the Beyond 50 cohort, rates of poor social health were high with a quarter of the population reporting that they were dissatisfied with their current social relations and more than one in six identifying as moderately to severely lonely. As social health has been recognised as a key factor that contributes to healthy ageing, this high prevalence could have a major impact on population health outcomes. Previous studies have identified that both social isolation and loneliness are associated with poor health, depression, poor quality of life and increased risk of dementia [45, 46]. Despite these recent insights into the negative health outcomes associated with poor social health, correlates and associated outcomes have been underexplored. Social health data collected from the Beyond 50 study will provide insights into the association between social isolation, loneliness and mental health, and how this influences individuals' health behaviours and quality of life to provide a more holistic understanding of psychosocial health and related outcomes within this population.

In contrast to other comparable Australian population studies, the Beyond 50 study has several key advantages to uncover insights into substance use patterns in older adults. While data collected by the Australian Bureau of Statistics National Health Survey [47] and National Drug Strategy Household Survey [48] collect data at regular time points, this is done as a repeated unlinked cross‐sectional survey and as such cannot be used to determine directionality of substance use related correlates. Other Australian cohort studies, such as the Household, Income and Labour Dynamics in Australia study [49] and ‘45 and Up’ [50], have limited capture of alcohol and other drug outcomes, while the 45 and Up study does not collect annual data, which limits their ability to detect nuances in the relationship between substance use and psychosocial health.

### 4.1 Strengths and Limitations

Strengths of the study include efforts to ensure representation of minority populations, including people of Aboriginal and Torres Strait Islander descent, at levels similar to the local population of the same age bracket. While the number of participants in the cohort is too small to enable specific analysis of these populations, their inclusion in the cohort allows us to capture their experiences and enriches the dataset. High survey completion rates allowed for inclusion of only completed survey data, which minimised attrition bias. However, for participants who started the survey but did not complete it, we have no way of determining if their experiences differed from those included in the cohort. High representation of people with high educational attainment and being married, while not representative of the broader population, may help to reduce attrition bias in subsequent waves of data collection as these characteristics are often associated with higher cohort retention in longitudinal studies.

The study uses self‐report measures, and some responses may be subject to recall bias and social desirability bias. However, self‐reported health has long been established to be a reliable measure of older adult morbidity [51, 52], and survey responses were confidential and deidentified to support participant disclosure. Some of the substance use subscales showed low internal consistency, but this may be limited due to the small number of items within the subscales. The planned linkage of survey results with healthcare data will allow self‐report to be complemented by externally verifiable data, such as the frequency of emergency department presentations. There were some differences between the cohort characteristics and the broader population, including gender distribution, marital status and educational attainment, which will need to be controlled for in analyses and considered when interpreting study findings. Those that were recruited via mailout were more likely to be married/in a de facto relationship or have a higher income. While academic studies have previously associated voting with higher rates of being married and having higher income [53, 54], the Australian Electoral Commission reports uniformly high turnout across demographic groups due to compulsory enrolment and voting requirements [55].

## 5 Conclusion

The characteristics of the Beyond 50 cohort demonstrate the vulnerability of Australian older adults to issues relating to poor psychosocial health and substance use. As such, findings from this unique cohort will provide rich information that can directly inform local and national health policy and may contribute to global knowledge of healthy ageing.

## Author Contributions

R.L. developed the first draft of the manuscript and revisions with all other authors' input. L.P. is a co‐author of the paper. P.D., T.L., A.L., N.E.A., S.A., K.D.H. and L.A. are co‐investigators of the study and were involved in study design and development. B.R. is a consultant biostatistician involved with Beyond 50 analysis plans. S.N. is the principal investigator of the study. All authors have read and approved the final manuscript.

## Funding

Funding was received through the Community Health and Hospitals Program Grant from the Department of Health and Aged Care, Commonwealth Government of Australia, for the National Centre for Healthy Ageing (Living Labs Round 3) and through the Ian Potter Foundation (Grant #31111278). S.N. and L.P. are the recipients of NHMRC Investigator Research Fellowships #2025894 and #2016909. R.L. is the recipient of PhD Scholarships from Monash University and the Monash Addiction Research Centre.

## Ethics Statement

Ethical approval for the prospective study was granted by the Peninsula Health Human Research Ethics Committee (No. 96756), and for the co‐design phase of the study by Monash University Human Research Ethics Committee (No. 35909).

## Conflicts of Interest

The authors declare no conflicts of interest.

## Supporting information


**Supporting Information: A** Strengthening the reporting of observational studies in epidemiology (STROBE) checklist.

**Supporting Information: B** Consumer and stakeholder involvement.

## Data Availability

The Beyond 50 research team welcomes collaboration with external parties to maximise the use of this rich data source, with the development of appropriate data sharing agreements and ethical approval. The key outcomes of the study focus on mental health, social health and substance use. However, there is the opportunity to add additional questions in future waves of data collection to explore other factors associated with healthy ageing. Interested researchers are encouraged to contact the research team which will manage data requests on a case‐by‐case basis. The data that supports the findings of this study will be made available on reasonable request with appropriate ethical approval and data security arrangements.
