# Supplementary material for: Characterising the Beyond 50 Cohort: Preliminary Exploration of Physical and Psychosocial Health Factors and Substance Use Among a Cohort of Community Dwelling Adults Aged 50–70 Years
Source: Drug Alcohol Rev. 2026 Aug 9;45(6):e70228. doi: 10.1111/dar.70228 (PMC13453339; doi:10.1111/dar.70228)
Supplement: Supplementary file 2 [file DAR-45-0-s002.docx]

**Supporting Information B**

**Consumer and stakeholder involvement**

**Codesign and consultation**

The *Beyond 50* study was designed with input from clinical and local service providers, academics, and members of the target audience (people aged 50-70 years living in the Frankston and Mornington Peninsula local government areas) through a series of focus groups, workshops and interviews to ensure that study outcomes would be relevant for informing local service provision.

Two workshops were conducted in February 2023, one face-to-face and one online, which were supplemented with individual interviews where participation in the workshops was not possible. Workshop participants included members from local council, Peninsula Health, the [Rehabilitation, Ageing and Independent Living (RAIL)](https://www.bing.com/ck/a?!&&p=4e5e4bd5783da463JmltdHM9MTcxNTQ3MjAwMCZpZ3VpZD0xNDY0MjUyYS04MDg2LTZiZmUtMWMyMC0zMTAyODE2YzZhNTQmaW5zaWQ9NTIxNw&ptn=3&ver=2&hsh=3&fclid=1464252a-8086-6bfe-1c20-3102816c6a54&psq=monash+RAIL&u=a1aHR0cHM6Ly93d3cubW9uYXNoLmVkdS9tZWRpY2luZS9zcGFoYy9yYWlsL2hvbWU&ntb=1) and Monash Addiction Research Centre (MARC) at Monash University, Edith Cowan University, Turning Point and the National Centre for Healthy Ageing (NCHA).

The consultation phase directly informed the development of the study’s research priorities and key aims, the final study name, and determined the optimal age range (which included participants from 50 years of age) so we could capture the experiences of populations that may age earlier in life, such as people of Aboriginal and Torres Strait Islander descent and people from some culturally and linguistically diverse backgrounds. Once research priorities were established, the research team identified relevant validated scales and measures and provided advice on how to reach the target population with specific concern on representation from areas of low sociodemographic advantage. A summary of the main outcomes of the codesign process are listed below:

*Research questions and key outcomes*

- Key outcomes were defined from the perspectives of the affected communities with lived experience, their caregivers and service providers. Two priority research questions were identified:
  1. What are the key drivers of mental health problems and substance use in older adulthood?
  2. Can we find ways to identify those at risk of poor outcomes (e.g. suicidal behaviours, substance use related harms) earlier to inform/target prevention interventions?
- The workshops also identified additional areas of interest, including potential differences between planned and unplanned retirement, and identifying potential protective or risk factors that impact substance use behaviours.

*Target population*

- The workshops provided additional advice on the target age group for the study. Capturing the experiences of vulnerable populations who experience premature ageing (e.g. Aboriginal and/or Torres Strait Islander communities, people that experience homelessness) was a key consideration. As a result, the decision was made for the to expand the age range to include individuals from the age of 50-70.

*Recruitment methods*

- Stakeholders identified a range of contacts and community groups within the geographical area, including cultural/ religious leaders, members of local council and community-based organisations such as local Men’s Sheds, Chatty Café’s, and sports clubs.
- Different recruitment media was suggested, including the use of local newsletters and newspapers, social media, and distribution of flyers to key community gathering places throughout the area (e.g. libraries, community centres, shopping centres). The need for multiple and varied forms of advertisement was highlighted during the workshops and interviews to promote recruitment of a diverse and representative cohort.
- An area of concern was representation from vulnerable populations, and geographically isolated locations within the local area. Examples provided by workshop attendees included culturally and linguistically diverse (CALD) populations, Aboriginal and Torres Strait Islander populations, transient populations, and areas of low socioeconomic status (SES).
- The workshops highlighted the need for plain, inclusive language to enable equitable access, and targeted recruitment strategies to ensure representation. One geographical area identified was Frankston North, an area of low SES which is often overlooked and underrepresented.

*Measures*

- Validated measures for inclusion were explored to confirm that they are acceptable and capture key outcomes that are important to those with lived experience, clinicians, community partners, and research experts.

*Additional outcomes*

- The final study name “*Beyond 50: Health and social outcomes in the Frankston and Mornington Peninsula”* was decided with input into a range of options through the codesign with the final name selected by popular vote of the codesign group.

**Advisory Board**

Based on advice from the stakeholders and consumers, a project advisory board was established which provides ongoing advice on the development of ongoing priorities for data analysis. Advisory board members include three ageing specialists, the founder of an assisted living service in the local area, two positive ageing local council representatives and a community member from the target population. The first advisory board meeting was held in February 2024 with an ongoing commitment to meet twice a year, with open correspondence in between when needed.

Specifically, the Advisory Board will provide advice on recruitment strategies and approaches to aid the formation and retention of the cohort, the development of priorities for specific analyses to inform policy and practice, how to effectively translate research findings across different sectors and how to maximise the policy relevance of current and planned work.
